# Sevoflurane Improves Hemorrhagic Shock and Resuscitation-Induced Cognitive Impairments and Mitochondrial Dysfunctions through SIRT1-Mediated Autophagy

**DOI:** 10.1155/2022/9771743

**Published:** 2022-03-11

**Authors:** Jianwei Shu, Xiaotong Huang, Qizhi Liao, Jianan Wang, Yuqi Zhou, Yihuan Chen, Ming Chen, Cheng Qian, Ye Zhang, Xianwen Hu, Chunxia Huang

**Affiliations:** ^1^Department of Anesthesiology, The Second Affiliated Hospital of Anhui Medical University, Hefei City, Anhui Province, China; ^2^Key Laboratory of Anesthesiology and Perioperative Medicine of Anhui Higher Education Institutes, Anhui Medical University, Hefei City, Anhui Province, China; ^3^Scientific Research and Experiment Center of the Second Affiliated Hospital of Anhui Medical University, Hefei City, Anhui Province, China; ^4^The Second Clinical Medical College of Anhui Medical University, Hefei City, Anhui Province, China; ^5^Center for Scientific Research of Anhui Medical University, Hefei City, Anhui Province, China

## Abstract

Cerebral ischemia reperfusion injury (IRI) induced by hemorrhagic shock and reperfusion (HSR) is the main cause of death following trauma. Previous studies indicated the neuroprotective effect of sevoflurane postconditioning (SP) in cerebral IRI. However, the mechanisms still remain elusive. Cerebral IRI models with SP were established by using HSR with C57BL/6 mice (male, 3-month-old) *in vivo* and by using oxygen glucose deprivation and reoxygenation (OGD/R) with HT22 cells *in vitro*. Postoperative cognition was evaluated by the Morris water maze, novel object recognition, and elevated plus maze tests. The role of SIRT1 was determined by using siRNA, a sensitive inhibitor (EX527), or an overexpression shRNA-GFP lentivirus. IRI caused significant disabilities of spatial learning and memory associated with enhanced cerebral infarct and neuronal apoptosis, which were effectively attenuated by SP. IRI also made a significant decrease of SIRT1 accompanied by oxidative stress, mitochondria dysfunction, and inactivated autophagy. SP or genetically overexpressing SIRT1 significantly suppressed defective autophagy, mitochondrial oxidative injury, and neuronal death caused by HSR or OGD/R. However, genetic suppression or pharmacological inhibition of SIRT1 significantly reversed the impact of SP treatment on mitochondrial DNA transcription ability and autophagy. Our results demonstrate that the loss of SIRT1 causes a sequential chain of mitochondrial dysfunction, defective autophagy, and neuronal apoptosis after IRI in the preclinical stroke models. Sevoflurane postconditioning treatment could effectively attenuate pathophysiological signatures induced by noxious stimuli, which maybe mediated by SIRT1.

## 1. Introduction

Approximately 1.9 million people die of hemorrhagic shock every year in the world [1]. Hemorrhagic shock reperfusion (HSR) injury occurs when restoring circulating blood volume with diluted hemoglobin concentration, which further reduced oxygen supply and aggravated tissue damage [2]. As a scenario of ischemia-reperfusion injury (IRI), HSR increases the long-term mortality, leaves serious sequelae [3, 4], such as cognitive dysfunction [5], and causes serious burden to patients' families and the society. Although early mortality is low after severe trauma, chronic critical illness is a common trajectory in survivors and is associated with poor long-term outcomes. Advancing age, shock severity, and persistent organ dysfunction are predictive variables of chronic critical illness [4]. Therefore, early identification may facilitate targeted interventions to change the trajectory of this morbid phenotype.

Silent information regulator-1 (SIRT1) is a nicotinamide adenine dinucleotide- (NAD+) dependent histone deacetylase, which can catalyze the deacetylation of acetyl lysine of histone and nonhistone substrates (such as p53 and FOXO). SIRT1 participates in the regulation of glucose and lipid metabolism, inflammation, cell aging and apoptosis, oxidative stress, and tumor formation. Numbers of studies reported that SIRT1 was downregulated in the IRI models, such as middle cerebral artery occlusion (MCAO) and reperfusion, oxygen glucose deprivation and reoxygenation (OGD/R). And it was also associated with multiple biocellular processes, including the activation of apoptosis and neuroinflammation [6], the suppression of angiogenesis [7], and mitochondrial oxidative stress and dysfunction [8]. In SIRT1^−/−^ animals, the area of cerebral infarction was significantly increased following focal cerebral ischemia [9]. SIRT1 gene overexpression significantly alleviated hippocampal damage, including less cerebral infarction, and reduced neuronal degeneration and cognition improvement [10]. It has been regarded that SIRT1 elicits neuroprotective effect in decreasing apoptosis and neuronal damage through regulating autophagy [11]. Autophagy is a cellular homeostatic program for the turnover of cellular organelles and proteins. Emerging evidence indicates autophagy as a critical modulator for cerebral IRI [12]. However, the association between SIRT1 and autophagy in IRI is still not fully understood.

In addition to anesthesia, sevoflurane can also alleviate cerebral IRI. In the early phase after transient global cerebral IRI, the activation of antioxidant enzymes (Nrf2/HO-1) may contribute to the neuroprotection of SP [13]. Furthermore, SP alleviates neuronal death and axon demyelination in neonatal rats by regulating microglia autophagy. Therefore, SP provides long-term cognitive, learning, and memory protection [14]. In our previous study, SP improved the impairments of spatial learning and memory induced by HSR in rats [15]. SP remarkably reduced the apoptosis of hippocampal neurons through reducing the endoplasmic reticulum stress. Moreover, SP stabilized the integrity of mitochondrial structure and function via preventing the opening of the mitochondrial mPTP channel [16]. However, the role of SIRT1 in the neuroprotective impact of SP has not been clearly illustrated.

In the present study, we aimed to investigate the mechanism by which SP potentially improved the cognitive deficits following HSR in mice. The cognition and anxiety-like behaviors were evaluated following SP without or with the SIRT1 inhibitor in HSR mice. The characteristics of mitochondrial morphology and function were indicated in OGD/R with either SP or SIRT1 siRNA transfection in HT22 cells. The association between SIRT1 and autophagy in the IRI was demonstrated in both HSR and OGD/R without or with SP.

## 2. Materials and Methods

### 2.1. Animals

Male C57BL/6 mice weighing 25-30 g were provided by Laboratory Animal Center in Anhui Medical University. All experimental procedures were performed in accordance with the NIH Guide for the Care and Use of Laboratory Animals and approved by the Ethics Committee for the use of experimental animals in Anhui Medical University (reference number LLSC20190765, date of approval 26/10/2019). Animals were randomly divided into specific groups depending on the different purposes. Mice were bred and housed in a temperature- and humidity-controlled room with a 12/12 h light/dark cycle. All animals had access to food and water ad libitum. One-week acclimatization period was applied before experiment employment. All behavioral tests have been performed during the light phase.

### 2.2. Hemorrhagic Shock and Resuscitation Mouse Model

Mice were fasted for 12 h before surgery and free to drink water. Hemorrhagic shock and resuscitation (HSR) were performed as described previously [17]. Polyethylene catheters were inserted into the right carotid artery and left jugular vein for blood extraction and infusion, respectively. Approximately 50% of the total blood volume (total blood volume [18] = weight (g) × 7%) were continuously extracted within 30 min through a two-way automatic infusion pump (Genie Touch, Kent Scientific Corporation, USA). After 1 hour, the collected blood was infused within 30 min at a constant speed. Rectal temperature was maintained at 37.0 ± 0.5°C by using a heating blanket. Finally, all catheters were removed, and the mouse was returned to its cage for recovery. The sham operation was only catheter intubation in the right carotid artery and left jugular vein without withdrawing or transfusing blood.

### 2.3. Intracerebroventricular Lentivirus Injection

To determine if the supplement of SIRT1 could reverse the impact of HSR, SIRT1 overexpression shRNA-GFP lentivirus (titer: 2.0 × 10^9^ TU/ml) and control lentiviral vector (LV-CTR) (titer: 1.5 × 10^9^ TU/ml) were purchased from GeneChem Company (Shanghai, China). After anesthesia, total 3 *μ*l control lentiviral vector or SIRT1 overexpression shRNA-GFP lentivirus was injected in the right lateral ventricle at the rate of 0.5 *μ*l/min. The following coordinates (relative to bregma) were used, -0.2 mm AP, -1 mm ML, and -2.5 mm DV according to the stereotaxic atlas of Paxinos and Franklin (Third edition, 2007) by using a stereotaxic apparatus (RWD Life Science Co., Ltd., China). To prevent reflux or backflow, the needle was slowly removed at 10 min after the injection. Seven days after lentivirus injection, mice were subjected to HSR.

To downregulate SIRT1, the inhibitor EX527 (i.p., 10 mg/kg) was administrated at 30 min before catheter intubation [19].

### 2.4. Behavioral Tests

#### 2.4.1. Morris Water Maze Test

According to the previous report [20], the apparatus (Zhenghua Biologic, China) was consisted of a circular swimming pool (120 cm diameter and 50 cm height), a depth of 30 cm covering black platform (10 cm diameter), and a camera with a video recording system on the ceiling to capture the movement of animals in the tank. The pool was divided into four quadrants with specific conspicuous visual cues. The platform was fixed in the center of the southwest and submerged approximately 0.7 cm below the water surface.

The spatial acquisition training was started on the 3rd day after HSR and carried out for 6 successive days. For each trial, mice randomly entered into each quadrant facing the wall. If the mouse failed to find the platform within 60 sec, it was guided to the platform and allowed to stay for 10 sec. The escape latency was recorded. To evaluate memory capability, the probe test was given at 24 hours of the last training trial. After removing the platform, mice were placed in the opposite quadrant searching for 60 sec [21]. The swimming distance (cm) and the proportion of time spent in the target quadrant (%) were documented through the computer image analyzer.

#### 2.4.2. Open Field Test

In an empty and light grey square arena (40 cm × 40 cm × 40 cm) (RWD Life Science, China), mice were placed in the center, and its behavior was recorded from 3 to 15 min [22]. The distance and the time spent in the center area were analyzed by PanLab Smart 3 software (Harvard, USA).

#### 2.4.3. Novel Object Recognition Test

The novel object recognition test consists of three phases with 24-hour interval: habituation, familiarization, and test phase. In our experience, the habituation phase is equal to the open field test. During the familiarization phase, a single mouse was placed in the open field arena containing two identical objects (A+A) for 10 min. During the test phase, the mouse was returned to the arena with two different objects: one is identical to the old and the other one is novel (A+B) [23]. The total exploration time and the exploration time of each object were recorded by PanLab Smart 3 software.

#### 2.4.4. Elevated plus Maze Test

To evaluate the changes of anxiety-related behaviors in mice, elevated plus maze (EPM) test was conducted on the 14th day after HSR. The apparatus consisted of plastic open and closed arms (5 cm width and 35 cm length). The closed arm was about 15 cm height, and the height of the maze from the ground was about 40-55 cm. The animals moved freely in the elevated cross maze (RWD Life Science, China) for 10 min. The moving distance and residence time in the arms were recorded and analyzed by PanLab Smart 3 software.

### 2.5. Infarct Volume Assessment

After EPM test, the animals were sacrificed, and the entire brain was fast frozen at -20°C for 20 min. Then, 1 mm thick coronal sections were continuously segmented and incubated in 2% 2,3,5-tribenzene tetrachloride (TTC) for 30 min at 37°C. Brain slices were further fixed in 4% polyformaldehyde for 24 hours and then digitally scanned to a computer for analysis using ImageJ software (National Institutes of Health, USA): Infarction volume% = (infarction area × 1 mm)/(whole brain area × 1 mm) × 100%.

### 2.6. Oxygen and Glucose Deprivation/Reoxygenation Protocol

The mouse hippocampal neuronal cell line (HT22) (Jennio Biotech Co., Ltd., Guangzhou, China) was cultured in Dulbecco's modified Eagle's medium (DMEM) (HyClone, USA) with 10% fetal bovine serum (FBS) at 37°C in a humidified atmosphere containing 5% CO_2_. When reaching 70-80% confluency, cells were subjected to OGD/R. Briefly, oxygen and glucose deprivation (OGD) was carried out in an anaerobic chamber (Stem Cell, USA) containing 95% N_2_ and 5% CO_2_. Cells were incubated with glucose-free DMEM and FBS for 4 hours at 37°C. After replacing the medium to the ordinary DMEM containing 10% FBS, cells were further cultured in the CO_2_ incubator for 4 hours.

To genetically inhibit the expression of SIRT1, siRNA transfection was introduced 24 hours before OGD/R without or with sevoflurane postconditioning. Nonsense sequence for the SIRT1 negative control (SIRT1-NC) and siRNA against SIRT1 (si-SIRT1) were purchased from GenePharma (Shanghai, China). According to the manufacturers' instructions, HT22 cells were seeded into 6-well plates and cultured to 50-60% confluence. Then transfection was performed with 20 *μ*M siRNA using 4 *μ*l of jetPRIME Transfection Reagent (Polyplus-Transfection®) in 200 *μ*l jetPRIME buffer. After 10 min, 2 ml fresh medium was added for further incubation. No-load siRNA plasmid was used as a control. The efficiency of SIRT1 intervention was confirmed by RT-PCR.

### 2.7. Sevoflurane Postconditioning In Vivo and In Vitro

At the onset of blood refusion, mouse was stabilized in a gas-tight anesthesia chamber with sevoflurane inhalation for 1 hour. Sevoflurane (Sevofrane®, AbbVie, Japan) was delivered by the carrier gases (5% CO_2_ and 95% O_2_, 3.5 l/min total gas flow) through the sevoflurane volatilizer (Vapor 2000, Germany). The concentration was maintained at 2.4% and measured by an anesthetic monitor (Mindray Bene View T8, Shenzhen, China). In the sham operation or HSR, mice were only exposed to the mixed gas (95% O_2_ and 5% CO_2_) for 1 hour.

For HT22 cells, at the beginning of reoxygenation with ordinary DMEM containing 10% FBS, cells were incubated in the gas-tight anesthesia chamber (Modular Incubator Chamber, Billups Rothenberg, USA) with 5% CO_2_ and 95% air. The percentage of sevoflurane in the chamber was maintained at 2 for 1 hour.

### 2.8. Evaluation of Cell Viability and Apoptosis

MTT (Beyotime Biotechnology, China) and CCK-8 (7-Seabiotech, China) assays were used to determine the viability of HT22 cells according to the manufacturer's instructions. Optical density was calculated with the absorbance measuring by Varioskan LUX microplate reader (Thermo Fisher Scientific, USA). The results were expressed as the ratio to the control values.

To detect apoptosis, HT22 cells were immunolabeled with cleaved caspase 3 (Cell Signaling Technology, USA) and identified with TdT-mediated dUTP nick-end labeling (TUNEL) (In Situ Cell Death Detection Kit TMR red, Roche, USA) according to the manufacturer's instructions. After fixation with 4% paraformaldehyde, HT22 cells were permeabilized with 0.1% Triton X-100 and then blocked with 10% normal goat serum for 2 hours at room temperature. HT22 cells were further incubated with primary antibody against cleaved caspase 3 overnight at 4°C and subsequent Alexa Fluor 488 secondary antibody (Invitrogen, USA) for 2 hours at room temperature. Finally, HT22 cells were exposed to TUNEL reagent for 1 hour at 37°C. The nucleus was recognized by DAPI (Sigma-Aldrich, USA), and the apoptotic cells were examined under a laser scanning confocal fluorescent microscope (Carl Zeiss LSM 880, Germany).

### 2.9. Assessment of Oxidative Stress and Mitochondrial Transmembrane Potential

Oxidative stress in the HT22 cells was measured using MDA and SOD assays (Nanjing Jiancheng, China) according to the manufacturer's recommendations. ATP content was also evaluated (Nanjing Jiancheng, China) according to the manufacturer's recommendations. MitoSOX Red (Invitrogen, USA) was used to measure the accumulation of mitochondrial ROS. JC-1 Mitochondrial Membrane Potential Dye (Invitrogen, USA) was used to evaluate mitochondrial transmembrane potential in HT22 cells. Briefly, HT22 cells were treated with MitoSOX Red or JC-1 solution at 37°C for 10-20 min and then detected by the fluorescent microscope (Axio Vert. A1, ZEISS, Germany). According the manufacturer's instructions, the average fluorescent intensities at 488 nm and 590 nm were determined by ImageJ software (National Institutes of Health, USA) and then analyzed as the red/green ratio.

### 2.10. Quantitative Real-Time Polymerase Chain Reaction

According to the manufacturer's protocol, total RNA from HT22 cells was extracted using TRIzol reagent (TAKARA, Japan). After reverse transcription with HiScript® III RT SuperMix (Vazyme, China), PCR was performed with StepOnePlus™ Real-Time PCR system (Applied Biosystems, USA) with the AceQ®qPCR SYBR Green Master Mix Kit (Vazyme, China). NADH dehydrogenase subunits 1 (ND1) and cytochrome c oxidase subunit 3 (COX3) were used to reflect the transcript level of mtDNA. Primers of SIRT1, ND1, and COX3 were purchased from Genepharma (Shanghai, China), and sequences were as follows: SIRT1 (F: 5′-GCAGGTTGCAGGAATCCAAA-3′, R: 5′-GGCAAGATGCTGTTGCAAAG-3′), ND1 (F: 5′-GGATCCGAGCATCTTATCCA-3′, R: 5′-GGTGGTACTCCCTCTGTAAA-3′), and COX3 (F: 5′- CGTGAAGGAAACTACCCAGG-3′, R: 5′-CGCTCAGAAGAATCCTGCAA-3′). The relative levels of SIRT1, ND1, and COX3 were normalized to internal control GAPDH with 2^-*ΔΔ*Ct^ method. The relative mRNA level of SIRT1 was shown in the SFig. 3.

### 2.11. SDS-PAGE and Western Blot Analysis

Total proteins were extracted and subjected to 10% or 15% polyacrylamide gels electrophoresis. Nonspecific binding sites were blocked with 5% nonfat dry milk for 1 hour at room temperature. Then, membranes were incubated with specific primary antibodies overnight at 4°C, including SIRT1 (Cell Signaling Technology, USA), Bax (CST, USA), Bcl-2 (CST, USA), Pink1 (CST, USA), Parkin (CST, USA), LC3B (CST, USA), mtTFA (NOVUS, USA), and GAPDH (Sigma-Aldrich, USA). After the incubation with horseradish peroxidase-conjugated secondary antibodies (ZSGB-BIO, Beijing, China) for 2 hours at room temperature, the immunoreactive band signal intensity was subsequently visualized by chemiluminescence (SuperSignal™ West Femto Maximum Sensitivity Substrate) (Thermo Fisher Scientific, USA). All immunoblots were normalized for gel loading with GAPDH (Sigma-Aldrich, USA) antibody. The intensities of chemiluminescent bands were measured by ImageJ software (National Institutes of Health, USA).

### 2.12. Deacetylase Assay

Nuclear proteins from the cells were firstly extracted using a nuclear and cytoplasmic protein extraction kit (Beyotime, Shanghai, China). Then, the NAD+-dependent deacetylase activity was evaluated by using a SIRT1/Sir2 deacetylase fluorometric assay kit Ver.2 (CycLex, Medical & Biological Laboratories Co., Ltd., Japan). According to the manufacturers' instructions and the preliminary experiments, the SIRT1 activity was expressed as the rate of reaction for the first 30 min, when there was a good linear correlation between the fluorescence and the reaction time.

### 2.13. Transmission Electron Microscopy

After specific treatments, HT22 cells were collected by centrifugation and then fixed overnight with 2.5% glutaraldehyde at 4°C. After rinse in PBS (0.01 M, PH 7.2) for 6 hours at 4°C, samples were further fixed in 1% osmium tetroxide for 1 hour at 4°C. Following dehydration with gradient alcohol, samples were incubated in propylene oxide and epoxy resin for 2 hours at room temperature and then embedded in epoxy resin for further ultramicrosectioning (70 nm in thickness, Leica UC-7 microtome). The area of mitochondria (10000x) was analyzed by using ImageJ software. The number of mitochondria autophagy bodies (10000×) was observed in at least 10 visual fields (including intact neuronal bodies) by a JEM1400 electron microscope (JEOL, Japan).

### 2.14. Immunofluorescence Staining

After the behavioral observation, the mouse was deeply anesthetized, and the brain was removed and postfixed in 4% paraformaldehyde for 72 hours. Following the dehydration by using graded series of saccharose and embedding with OCT, coronal 16 *μ*m thick sections (from -1.46 mm to -2.46 mm posterior to bregma) were made. Nonspecific binding was blocked by 10% normal goat serum. Sections were incubated with primary antibodies against SIRT1 (CST, USA), LC3B (CST, USA), TOM20 (CST, USA), 8-OHdG (Santa Cruz Biotechnology, USA), and VDAC (CST, USA) overnight at 4°C. Alexa Fluor 568 or 488 secondary antibodies (Invitrogen, USA) were further incubated for 2 hours at room temperature. The nucleus was identified with DAPI. *Z*-stack images were acquired by using a laser scanning confocal fluorescent microscope (20x, 40x oil, and 63x oil immersion objectives) (Carl Zeiss LSM 880, Germany) equipped with ZEN light software at 1,024 × 1,024 resolution. All quantitative analyses were performed from at least three independent experiments. Data were analyzed with ImageJ software.

### 2.15. Statistical Analysis

The data of the Morris water maze were analyzed by two-way analysis of variance (ANOVA) followed by the Tukey *post hoc* test for repeated measures, using the statistic software GraphPad Prism 8.0 (Graph Pad Software Inc., USA). Other data, such as elevated plus maze test, normalized band intensities in western blot, and quantification of immunoreactivity, were analyzed by one-way ANOVA followed by the Tukey *post hoc* test. Normality of the data and homogeneity of group variances were assessed using the D'Agostino-Pearson omnibus normality test, Shapiro-Wilk normality test, and Kolmogorov-Smirnov test, respectively. Statistical significance was determined if *p* < 0.05. Data are represented as the mean ± SD in the context.

## 3. Results

### 3.1. Sevoflurane Postconditioning Improved Cognitive Deficits and Anxiety Induced by Hemorrhagic Shock and Resuscitation

**Figure 1:**
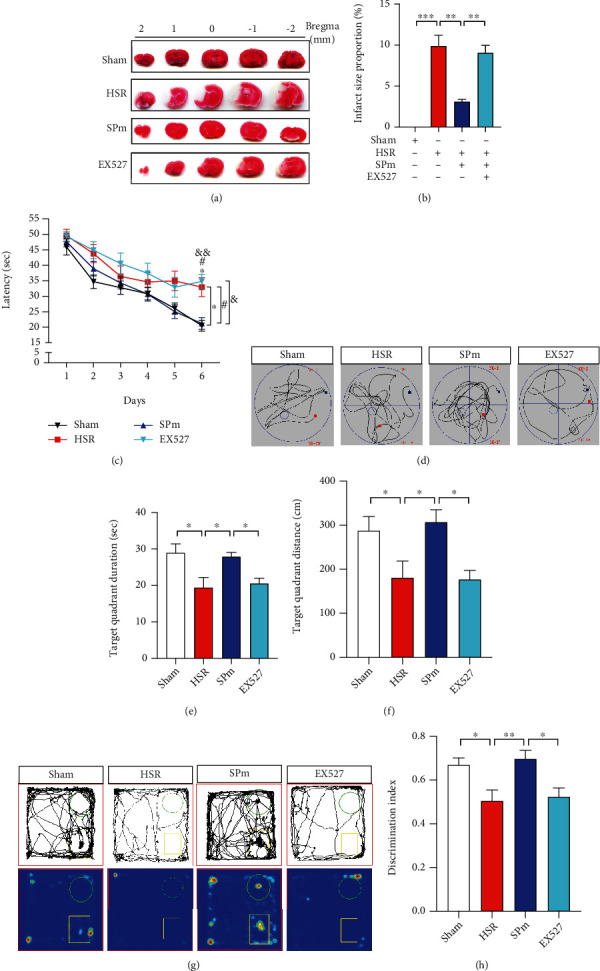
Sevoflurane postconditioning improved cognitive impairments induced by hemorrhagic shock and resuscitation (HSR). (a, b) Representative cerebral TTC staining photographs and the calculation of infarct size were made after HSR without or with sevoflurane postconditioning (SPm). EX527 (SIRT1 inhibitor) (i.p., 10 mg/kg) was administrated at 30 min before catheter intubation; then, mice were exposed to HSR with SPm. Infarct size of coronal brain sections was determined by TTC staining on postoperative day 14. HSR caused a significant cerebral infarction (white area). (c) A learning curve was made in the training session for spatial acquisition in the Morris water maze (MWM) test from postoperative day 3 to 8. (d–f) In the MWM test, the performance in the probe test included swimming route, duration, and distance in the target quadrant in the probe test. (g, h) Representative exploration route for two different objects, heat map, and discrimination index were recorded in the novel object recognition (NOR) test. The discrimination index was calculated by the ratio of time exploring the new object versus two different objects. All values are represented as mean ± SEM. *n* = 12 per group; ^∗^*p* < 0.05, ^∗∗^*p* < 0.01, and ^∗∗∗^*p* < 0.001, the HSR group vs. sham group; ^#^*p* < 0.05, the SPm group vs. HSR group; and ^&^*p* < 0.05, the EX527 group vs. SPm group.

We firstly established and tested the animal model of ischemia-reperfusion injury (IRI) in mice. On postoperative day 14, hemorrhagic shock and resuscitation (HSR) caused severe cerebral infarct. The introduction of sevoflurane postconditioning (SP) at the beginning of reperfusion significantly reduced cerebral infarct size. However, when pretreated with EX527 before SP, the infarct size was significantly stronger than SPm group (*F* = 33.82, Figures 1(a) and 1(b)).

To evaluate the impact of HSR on the spatial learning and memory, the Morris water maze (MWM) test was performed from postoperative day 3 to 9. Especially on 6th training day, mice in the HSR group acquired longer escape latency than mice in the sham group. After SPm treatment, the escape latency was remarkably shortened. Nevertheless, mice with EX527 pretreatment needed longer time to find the platform (*p*_time_ < 0.0001, *p*_group_ = 0.0104, and *p*_time∗group_ = 0.0189; Figure 1(c)). In the probe test for spatial memory determination, comparing with the sham group, mice after HSR swam less time (19.4 ± 9.6 sec vs. 29.0 ± 8.4 sec) and shorter distance (180.8 ± 131.7 cm vs. 287.4 ± 111.0 cm) in the target quadrant. SP significantly improved animal's spatial memory, which was indicated by swimming longer and farther. However, this improvement was effectively reversed by a single preadministration of EX527 (Figures 1(d)–1(f)).

In the novel object recognition (NOR) test, the discrimination index (DI) of mice after HSR (0.51 ± 0.17) suggested that mice spent equal time on exploring two different objects. Higher DI following SPm implied that it strengthened animals' discrimination ability to two different objects (0.70 ± 0.14). However, the addition of EX527 to SPm made a significant DI decline (*F* = 5.825, Figures 1(g) and 1(h)).

**Figure 2:**
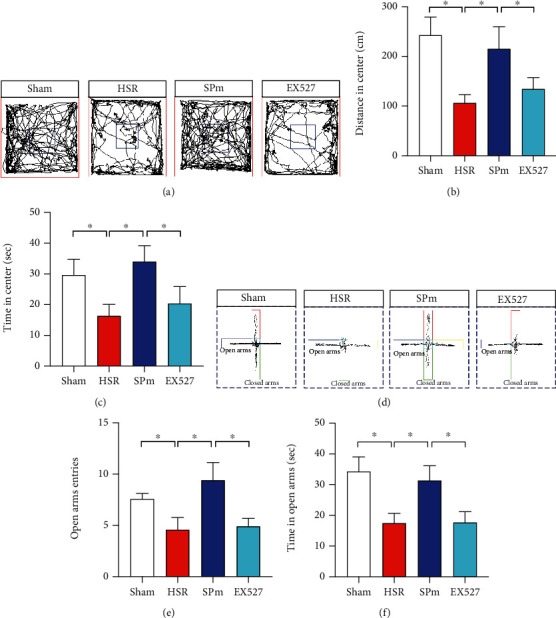
Sevoflurane postconditioning improved anxiety-like behaviors caused by HSR. (a–c) In the open field test (OFT), representative moving tracks in the arena were traced and descripted during their spontaneous activities. Particularly, moving distance and duration in the center area were further calculated as the signatures of anxiety. (d–f) In the elevated plus maze (EPM) test, representative moving tracks in both open and closed arms were traced and descripted within 10 min. To determine the anxious reactions, the entry frequency and the duration in the open arms were calculated. All values are represented as mean ± SEM. *n* = 12 per group, ^∗^*p* < 0.05.

To investigate the impact of SP on anxiety-like behaviors, open field test (OFT) and elevated plus maze (EPM) test were initiated in HSR mice without or with SP. In the OFT, mice following HSR were less active, evidenced by shorter moving distance and less residence time in the center area. In the SPm group, there were more spontaneous activities in the center area compared with the HSR group. When mice received EX527, they performed significantly inactive in the center area (Figures 2(a)–2(c)). In the EPM test, mice in the HSR group performed anxiety-like behaviors, such as fewer entries and less residence time in the open arms. The addition of SPm significantly relieved anxiety, which indicated by higher entry frequency (Figures 2(d) and 2(e)) and longer duration (Figure 2(f)) in the open arms. However, significant less open arm entries and duration were observed in the mice with EX527 pretreatment (*F* = 4.008, *F* = 4.624).

### 3.2. Sevoflurane Postconditioning Reversed SIRT1 Loss and Cell Apoptosis Caused by IRI In Vivo and In Vitro

**Figure 3:**
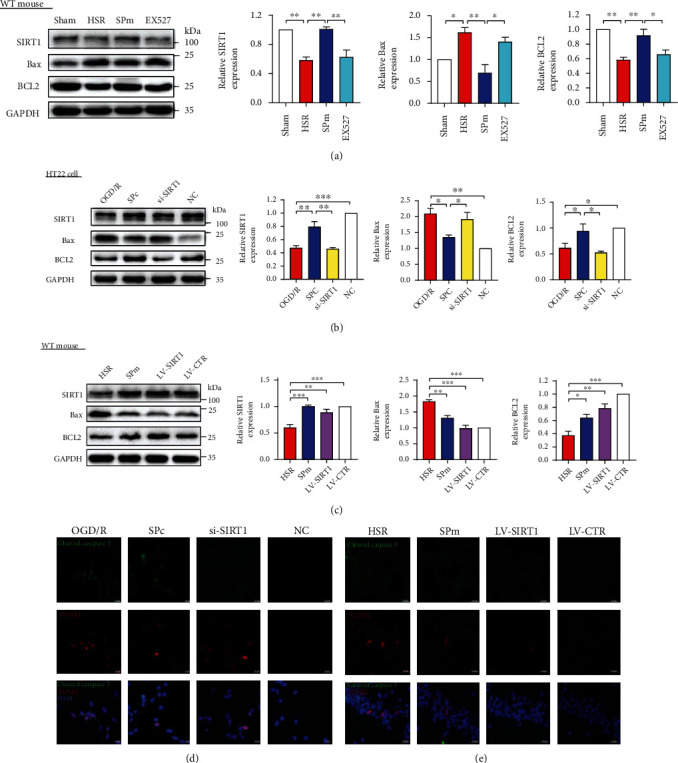
Sevoflurane postconditioning attenuated SIRT1 loss and cell apoptosis induced by ischemia-reperfusion injury in both *in vivo* and *in vitro* models. (a, b) By using western blotting analysis, protein expression levels of SIRT1, proapoptotic Bax, and antiapoptotic BCL2 were demonstrated following HSR or oxygen glucose deprivation and reoxygenation (OGD/R) with or without sevoflurane postconditioning (SP). After pretreatment with SIRT1 selective inhibitor EX527 or si-SIRT1 before SP, the levels of SIRT1 and apoptosis-related proteins were also measured. Nonsense sequence for SIRT1 was the negative control (NC). (c) After overexpressing SIRT1 through lentivirus transfection, protein levels of SIRT1, Bax, and BCL2 were evaluated in mice following HSR without SP. Control lentiviral vector (LV-CTR) was cerebrally injected without or with HSR or SPm. (d, e) Apoptotic cells induced by HSR or OGD/R were presented by double labelling with cleaved caspase 3 and TUNEL. All values are represented as mean ± SEM. *n* = 4‐6 per group; ^∗^*p* < 0.05, ^∗∗^*p* < 0.01, ^∗∗∗^*p* < 0.001, and ^∗∗∗∗^*p* < 0.0001. Scale bar = 20 *μ*m.

By using western blot analysis, consistent loss of SIRT1 was observed in IRI models *in vivo* and *in vitro*, including HSR, oxygen glucose deprivation, and reoxygenation (OGD/R) (0.59 ± 0.09-fold of Ctrl in mice; 0.46 ± 0.07-fold of Ctrl in HT22 cell, Figures 3(a) and 3(b)). Meanwhile, significant apoptosis was also observed with the increase in Bax expression and decrease in BCL2 expression (Figures 3(a) and 3(b)). After SP, changes of SIRT1, Bax, and BCL2 induced by HSR or ODG/R were significantly reversed. In addition, the deposition and distribution of SIRT1, as well as the deacetylation activity of SIRT1, were varied following OGD/R without or with SP (SFig. 1, SFig. 2). Either EX527 or si-SIRT1 significantly reduced SIRT1 expression accompanying with distinct more apoptosis (Figures 3(a) and 3(b), SFig. 3). When overexpressing SIRT1 by using lentivirus transfection before HSR, SIRT1 loss and apoptosis were significantly eliminated (Figure 3(c)).

Consistent with the distinct apoptosis, cell viability was remarkably dropped after OGD/R (0.49 ± 0.03-fold of Ctrl, SFig. 4). In addition to promoting cell survival, SP prevented apoptosis and DNA damage, which was evidenced with the decreased immunoreactivities of cleaved caspase 3, TUNEL, and 8-OHdG (Figures 3(d) and 3(e), SFig. 5 & 6). However, these decreases were significantly reversed after SIRT 1 gene silencing by using si-SIRT1 transfection in HT22 cells. When HSR mice were preinjected with SIRT1 overexpression lentivirus, less distributions of cleaved caspase 3^+^ and TUNEL^+^ immuno-signals were observed in the hippocampal CA1 region.

### 3.3. Sevoflurane Postconditioning Improved Oxidative Stress and Mitochondrial Dysfunction Caused by IRI In Vitro

**Figure 4:**
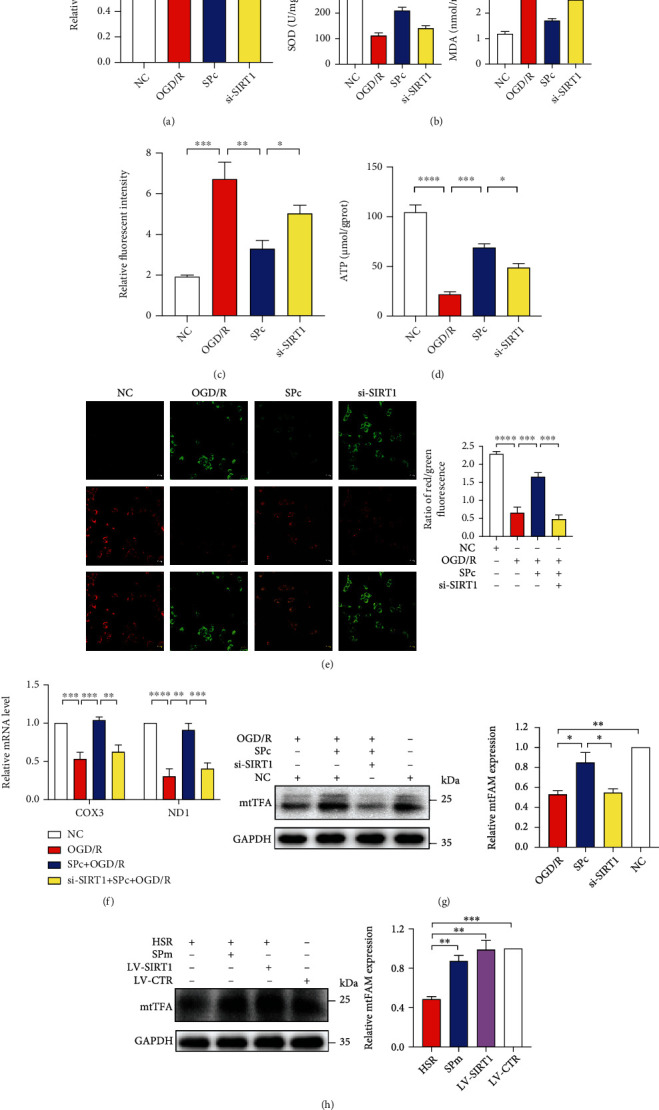
Sevoflurane postconditioning alleviated oxidative stress and mitochondrial dysfunction caused by OGD/R. (a) During OGD/R, sevoflurane postconditioning (SPc) increased cell viability in the CCK8 assays. Transfection of siRNA against SIRT1 (si-SIRT1) was introduced 24 hours before OGD/R with SPc. Nonsense sequence for SIRT1 was the negative control (NC). (b–d) SPc relieved oxidative stress with more SOD, less burdens of MDA and MitoSOX, and more ATP production. (e) Representative photographs and the quantification of mitochondria membrane potentiation (MMP) was determined by JC-1 assay in HT22 cells following OGD/R with SP treatment. Scale bar = 20 *μ*m. (f) Relative mitochondria DNA copy numbers of COX3 and ND1 were quantified by RT-qPCR after SPc with si-SIRT1 treatments. (g, h) Relative mitochondrial transcription factor A (mtTFA) protein expressions in HT22 cells and hippocampi following IRI with SIRT1 down- and upregulation. All values are represented as mean ± SEM. *n* = 4‐6 per group; ^∗^*p* < 0.05, ^∗∗^*p* < 0.01, ^∗∗∗^*p* < 0.001, and ^∗∗∗∗^*p* < 0.0001.

By using CCK8 assay, more survived cells were observed after SPc treatment than OGD/R (Figure 4(a)). In the HT22 cells, OGD/R made a remarkable oxidative stress, signed by the decrease of SOD production, increase of MDA accumulation (Figure 4(b)), and increase of MitoSOX immunoreactivity (Figure 4(c)). In addition, OGD/R dramatically reduced ATP production and disrupted mitochondrial membrane potentiation (MMP) determined by JC-1 assay (Figures 4(d) and 4(e)). SPc significantly alleviated oxidative stress with the evidence including more SOD yields, less MDA release, and less MitoSOX toxicity. SPc also promoted ATP production and improved mitochondrial damage through enhancing MMP. Otherwise, the effects of SPc on oxidative stress and MMP were completely reversed by genetic silence of SIRT1 with si-SIRT1.

To further investigate the transcript level of mtDNA during IRI without or with SPc, mitochondrial copy number including NADH dehydrogenase subunits 1 (ND1) and cytochrome c oxidase subunit 3 (COX3) was determined. OGD/R strikingly suppressed mitochondrial DNA transcript ability, which was boosted by SPc (Figure 4(f)). Additionally, both OGD/R and HSR decreased the expression of mitochondria transcription factor A (mtTFA). After SP, the increases of mtTFA were noted in both HT22 cells and mouse hippocampi. However, there was a prominent drop of mtTFA after SIRT1 gene silencing. The overexpression of SIRT1 by lentivirus transfection made a comparable level of mtTFA to SPm (Figures 4(g) and 4(h)).

### 3.4. Sevoflurane Postconditioning Reversed Autophagy Caused by IRI In Vitro

**Figure 5:**
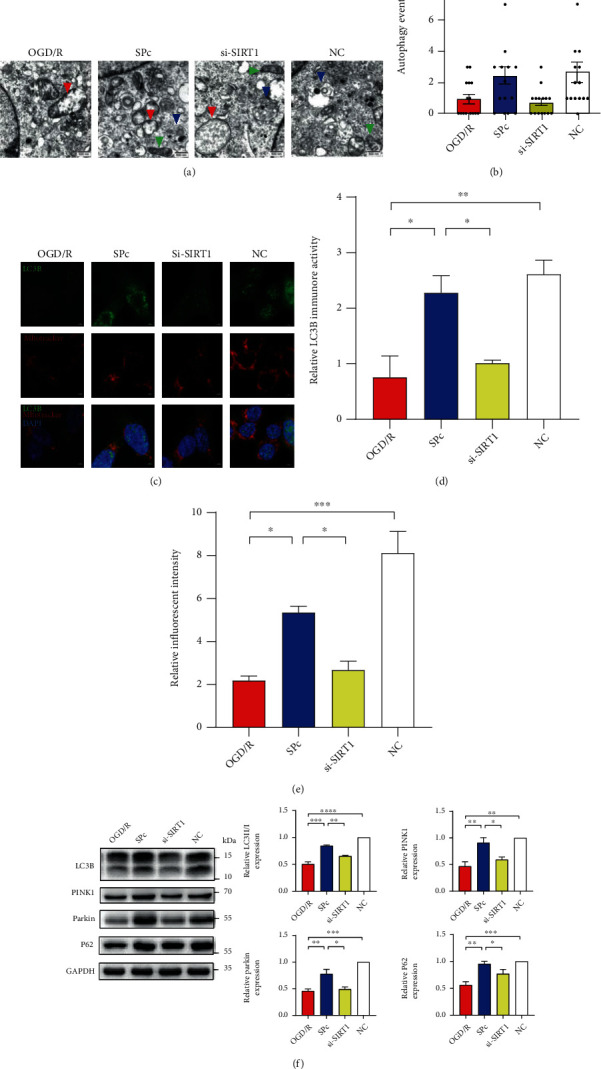
SIRT1 silence reversed the effect of sevoflurane postconditioning on autophagy during OGD/R. (a) Mitochondrial morphological changes under TEM following OGD/R and SPc. Green arrow, healthy mitochondria; red arrow, injured mitochondria; blue arrow, mitophagy. Scale bar = 500 nm. Sevoflurane postconditioning was performed at the beginning of reoxygenation in HT22 cells (SPc). During OGD/R, transfection of siRNA against SIRT1 (si-SIRT1) was introduced 24 hours before OGD/R with SPc. Nonsense sequence for SIRT1 was the negative control (NC). (b) By using electron microscopy images, autophagy-like events were quantified in HT22 cells after OGD/R and SPc, *n* = 4 neurons from 3 mice per each group. (c–e) The deposition and distribution of LC3B in HT22 cells were quantified after OGD/R and SPc. Scale bar = 5 *μ*m. Mitochondria were labelled with MitoTracker. The relative fluorescent intensity of MitoTracker was quantified. (f) Relative levels of autophagy-related proteins were determined in HT22 cells after OGD/R with SPc. All values are represented as mean ± SEM. *n* = 4‐6 per group; ^∗^*p* < 0.05, ^∗∗^*p* < 0.01, ^∗∗∗^*p* < 0.001, and ^∗∗∗∗^*p* < 0.0001.

Under TEM observation, OGD/R made mitochondria swelling and hypertrophy, as well as mitochondrial ridge disruption (Figure 5(a)). Meanwhile, less mitophagy events were noted (Figure 5(b)). The effect of SPc or si-SIRT1 on autophagy was further determined by immunofluorescent staining of LC3B (Figures 5(c)–5(e)). Furthermore, the levels of autophagy markers, such as LC3B, PINK1, Parkin, and P62, were significantly reduced following OGD/R in HT22 cells (Figure 5(f)). SPc successfully prevented mitochondria against swelling, ridge disruption, and vacuolization. SPc also improved the suppression of LC3B, PINK1, and Parkin induced by OGD/R. Otherwise, all these changes were reversed after the introduction of si-SIRT1 to SPc.

### 3.5. Sevoflurane Postconditioning Manipulated Autophagy through SIRT1 Intervention with Pharmacological Inhibition or Genetic Overexpression *In Vivo*

**Figure 6:**
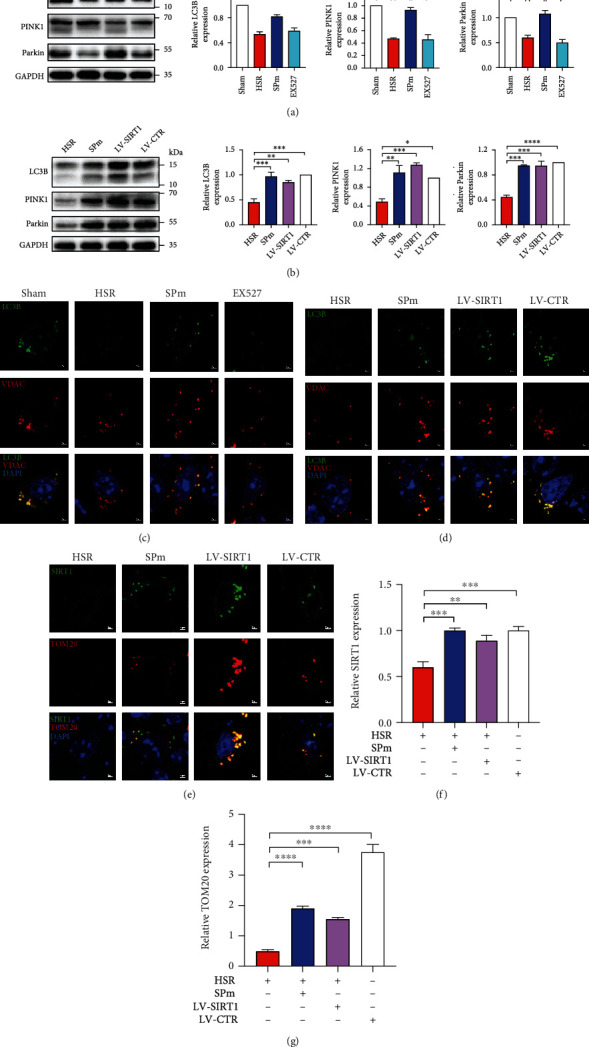
Regulation of autophagy and SIRT1 manipulation by sevoflurane postconditioning during HSR. (a, b) By using western blot analysis, relative levels of autophagy-related proteins were quantified in the hippocampi after HSR with SPm. Sevoflurane postconditioning was performed at the onset of blood refusion in mice (SPm). EX527 (SIRT1 inhibitor) (i.p., 10 mg/kg) was administrated at 30 min before catheters intubation; then, mice were exposed to HSR with SPm. After overexpressing SIRT1 through lentivirus transfection, mice were exposed to HSR without SPm. Control lentiviral vector (LV-CTR) was cerebrally injected without HSR or SPm. (c, d) The localization of LC3B was presented in the hippocampal CA1 region following SPm with pharmacological inhibition or genetic overexpression of SIRT1. (e–g) The quantification of SRIT1 and TOM20 in the hippocampus was presented following HSR without and with genetic overexpression of SIRT1. All values are represented as the mean ± SEM. *n* = 4‐6 per group. ^∗^*p* < 0.05, ^∗∗^*p* < 0.01, ^∗∗∗^*p* < 0.001, and ^∗∗∗∗^*p* < 0.0001.

Firstly, the impact of SP on autophagy in IRI was consistently demonstrated in the animal model of HSR (Figures 6(a) and 6(b)). After the systemic administration of EX527, there were noted decreases of LC3B, PINK1, and Parkin (Figure 6(a)). The effect of SPm without or with SIRT1 intervention on autophagy was also presented by the varied distributions of LC3B in the hippocampal CA1 region (Figure 6(c)).

To further investigate the effect of SIRT1 on autophagy, SIRT1 overexpression lentivirus was ventricularly injected one week before HSR. SPm and LV-SIRT1 comparably increased the protein expressions of LC3B, PINK1, and Parkin, as well as the distribution of LC3B in the hippocampal CA1 region (Figures 6(b) and 6(d)). Furthermore, significant deposition of SIRT1 was noted in the same region, associated with remarkable colocalization with TOM20 (Figures 6(e)–6(g)).

## 4. Discussion

We successfully demonstrated the neuroprotective effect of sevoflurane postconditioning (SP) in two ischemia reperfusion injury (IRI) models, hemorrhagic shock and resuscitation (HSR), and oxygen and glucose deprivation (OGD/R). SP significantly prevented the impairments of spatial learning and memory, as well as anxiety-like behaviors. It also improved the oxidative damage, mitochondrial dysfunction, and defective autophagy induced by IRI. However, the impact of SP on IRI was consistently reversed by the posttranslational modification of SIRT1 by pharmacological inhibitor or gene silencing.

The mechanisms of IRI are mainly related to oxidative stress, calcium imbalance, mitochondrial injury, excessive inflammatory response, endoplasmic reticulum stress, and programmed cell death. In the common animal model of cerebral IRI, such as middle cerebral artery occlusion (MCAO), distinct cerebral infarction and cognitive dysfunction are usually presented with apoptosis, neuroinflammation, oxidative stress, and mitochondrial dysfunction [8–11, 24]. In this study, HSR was used as another mouse model of IRI. It caused significant cerebral infarctions, spatial learning, and memory deficits, as well as object recognition disability. Meanwhile, mice after HSR also performed anxiety-like behaviors, including less active and more anxiety.

On the other hand, oxygen glucose deprivation and reoxygenation (OGD/R), as an *in vitro* model of IRI, suppressed cell viability with increased oxidative stress injury in HT22 cells. Mitochondria are a major target in hypoxic/ischemic injury. At the early stage of reperfusion, there is a promotion in the opening of mitochondrial membrane permeability transition pore (mPTP) [25] and increases in the releases of cytochrome c and apoptosis inducing factor. At a long period following ischemia-reperfusion, a large number of ROS interrupts the activities of respiratory chain complexes I, II, III, and IV. And the dysfunction of mitochondrial respiratory chain further increases the production and accumulation of ROS, triggering a series of cell damage [26], such as mitochondrial DNA (mtDNA) oxidative damage [27]. The mtDNA damage directly decreased the encoding of respiratory chain related proteins, which contributed to the dysfunctions of oxidative respiratory chain and ATP production. Consequently, ROS were further increased, so that oxidative damage was aggravated [28].

Disturbance of mitochondria function resulted in neuronal apoptosis and neurological dysfunction after cerebral infarction. In this study, consistent cell apoptosis was observed in HT22 cells after OGD/R and in the hippocampal CA1 region after HSR, respectively. In addition to the disrupted mitochondrial membrane potentiation and ATP generation, OGD/R also interrupted mitochondrial DNA (mtDNA) transcription. Because mtDNA is close to the respiratory chain and lacks histone protection, it is more vulnerable to the oxidative stress [29, 30]. Consistent to the findings in the aging process [27], we found that cerebral IRI made mtDNA oxidative damage, such as decreases in NADH dehydrogenase subunits 1 (ND1) and cytochrome c oxidase subunit 3 (COX3). Studies have shown that mtDNA oxidative damage can be detected 1 hour after IR, and cell death can be observed 6 hours later [31], suggesting that mtDNA oxidative damage may be one of the factors leading to early IR injury. Mitochondrial transcription factor A (mtTFA) is essential for mitochondrial biogenesis and plays a critical role in the maintenance of mitochondrial DNA replication and transcription [32]. However, mtTFA expression was consistently decreased in both HSR and OGD/R.

SIRTs are class III histone deacetylases and sorted into groups I–IV (SIRT1–3, SIRT4, SIRT5, and SIRT6/7). SIRT1 impacts multiple processes including chromatin remodeling, DNA repair, cell survival, and neurogenesis. It is essential for the maintenance of brain integrity, normal learning, memory, and synaptic plasticity in animals [33]. At 48 hours after focal ischemia, larger infarct volumes were displayed in SIRT1^−/−^ mice than the wild-type counterparts [9]. In contrast to SIRT1, SIRT2 and SIRT3 show diverse force in IR. At the early phase of ischemic stroke, SIRT2 mediates myelin-dependent neuronal dysfunction. In both mild and severe MCAO, there were no significant differences in neither infarct volumes nor inflammatory cell count between SIRT2^−/−^ and wild-type mice. Especially at 48 hours after reperfusion, SIRT2^−/−^ mice showed less neurological damage in the experimental stroke models [34]. It also reported that a compensatory rise in SIRT1 rather than the loss of SIRT3 after stroke contributed to the significant neuroprotective effect against IR or stroke injury in SIRT3^−/−^ mice [35]. By using two experimental IRI models (HSR and OGD/R), we found a significant decrease of SRIT1 associated with cell apoptosis and mitochondrial oxidative damage, which were strikingly improved after SIRT1 genetic overexpression or SP treatment.

In response to hypoxic-ischemic conditions, SIRT1 has a pivotal role in maintaining mitochondrial homeostasis by activating many downstream targets, such as regulating peroxisome proliferator-activated receptor *γ* coactivator *α* (PGC-1*α*) for mitochondrial biosynthesis, increasing the expression of ROS degrading enzymes [36], inhibiting p53 induced apoptosis through deacetylation [37], and regulating molecular mediators such as hypoxia-inducible factor 1*α* (HIF-1*α*), c-MYC, and AMPK [38], as well as SIRT1/2/3-Foxo3a-MnSOD/PGC-1 signaling pathways [39]. SIRT1 also plays a critical role in the turnover of defective mitochondria through mitophagy [40]. To promote neuronal survival in cerebral ischemia, mitophagy is initiated to eliminate damaged mitochondria through autophagy [12]. SIRT1 activation and autophagy induction contribute to the anti-inflammatory and neuroprotective activities of urolithin A in brain cells (BV2 microglia and APPSwe-transfected ReNcell VM human neural cells) exposing to lipopolysaccharide. Those changes are reversed in the presence of EX527 (SIRT1 inhibitor) and chloroquine (autophagy inhibitor) [41]. In our study, both SIRT1 and autophagy were suppressed by HSR and OGD/R and associated with cognitive dysfunction, mitochondrial dysfunction, and neuronal apoptosis. The overexpression of SIRT1 by shRNA-GFP lentivirus successfully improved the pathological disruptions induced by HSR.

During starvation, LC3 cycles between nucleus and cytoplasm depending on its nuclear deacetylation by SIRT1. Consistent to other cases [42, 43], we also found the dominant distribution of LC3 in the nucleus of intact hippocampal neuronal cells. However, IR suppressed the redistribution of LC3, which was partially attributed to SIRT1 loss. In addition to senescence and aging [44], translocation of SIRT1 from its original nuclear resides to cytosolic puncta was also found in IR in this study. The colocalization of SIRT1 and LC3 supports the new identify of SIRT1 as a new substrate of nuclear autophagy. SIRT1 degradation is mediated by LC3 with the involvement of nucleus-to-cytoplasm shuttling of SIRT1 and autophagosome-lysosome degradation [43, 45]. Interestingly, although SIRT1 prevents DNA damage and oxidative stress through the induction of autophagy, SIRT inhibits corticosterone-induced autophagy with subsequent enforced cell apoptosis [46]. Therefore, a further study is needed to clarify the relationship between SIRT1 and LC3 in the development of IRI.

Even though the effect of sevoflurane on cognition were controversial, such as the deleterious impact of sevoflurane exposure in the neonatal period [47], numbers of studies reported the protective effect of SP in varied IRI models. In myocardial IRI, SP reduces the production of mitochondrial ROS, increases the content of mitochondrial ATP, and thus reduces apoptosis through activating the JAK2-STAT3 pathway [48]. For transient global cerebral ischemia in rats, SP plays an anti-inflammatory role by blocking the TLR-4/NF-*κ*B pathway [49]. SP also attenuates OGD/R-induced autophagosome accumulation in the SHSY-5Y cell line [50]. Our previous studies demonstrated that SP prevented cerebral damage against HSR in rats [15, 16]. SP efficiently improved the spatial learning and memory ability by stabilizing the integrity of mitochondrial structure and function. Furthermore, the downregulation of endoplasmic reticulum stress also involved in the attenuated hippocampal neuronal apoptosis induced by SP through the IRE1*α*/caspase-12 pathway [16]. In this study, the employment of 1-hour SP dramatically prevented the cognitive and mental disorders induced by HSR in mice, as well as mitochondrial dysfunction and neuronal apoptosis attributed to OGD/R in HT22 cells. Pharmacological inhibition with EX527 or genetic suppression with siRNA effectively reversed the neuroprotective actions of SP in cognitive dysfunction, mitochondrial oxidative damage, and autophagy disturbance. Otherwise, it is notable to investigate if SP-induced neuroprotection could be overturned when autophagy was suppressed with specific inhibitors.

## 5. Conclusions

In this study, we showed that SP prevented the cognitive dysfunction and anxiety caused by HSR. SP improved neuronal apoptosis by attenuating mitochondrial oxidative stress and improving autophagy. SIRT1 potentially plays an essential role in the neuroprotective effect of SP against IRI. Our findings suggest that SIRT1 may be a functional target in modulating the oxidative stress and autophagy in IRI.

## Data Availability

The data used to support the findings of this study are available from the corresponding authors upon request.

## Supplementary Materials

Supplementary Materials Figure S1: sevoflurane postconditioning varied the deposition and distribution of SIRT1 following IRI. Figure S2: relative deacetylation activity of SIRT1 after sevoflurane postconditioning in HT22 cells. Figure S3: relative mRNA level of SIRT1 after siRNA transfection in HT22 cells. Figure S4: sevoflurane postconditioning promoted the cell viability following OGD/R. Figure S5: sevoflurane postconditioning attenuated the apoptosis induced by OGD/R. Figure S6: sevoflurane postconditioning attenuated DNA damage induced by OGD/R.
