# Supplementary material for: Sevoflurane Improves Hemorrhagic Shock and Resuscitation-Induced Cognitive Impairments and Mitochondrial Dysfunctions through SIRT1-Mediated Autophagy
Source: Oxid Med Cell Longev. 2022 Mar 11;2022:9771743. doi: 10.1155/2022/9771743 (PMC9068312; doi:10.1155/2022/9771743)
Supplement: Supplementary Materials — Figure S1: sevoflurane postconditioning varied the deposition and distribution of SIRT1 following IRI. Figure S2: relative deacetylation activity of SIRT1 after sevoflurane postconditioning in HT22 cells. Figure S3: relative mRNA level of SIRT1 after siRNA transfection in HT22 cells. Figure S4: sevoflurane postconditioning promoted the cell viability following OGD/R. Figure S5: sevoflurane postconditioning attenuated the apoptosis induced by OGD/R. Figure S6: sevoflurane postconditioning attenuated DNA damage induced by OGD/R. [file 9771743.f1.docx]

**Supplementary Materials**

Figure S1. Sevoflurane postconditioning varied the deposition and distribution of SIRT1 following IRI.

Figure S2. Relative deacetylation activity of SIRT1 after sevoflurane postconditioning in HT22 cells.

Figure S3. Relative mRNA level of SIRT1 after siRNA transfection in HT22 cells.

Figure S4. Sevoflurane postconditioning promoted the cell viability following OGD/R.

Figure S5. Sevoflurane postconditioning attenuated the apoptosis induced by OGD/R.

Figure S6. Sevoflurane postconditioning attenuated DNA damage induced by OGD/R.


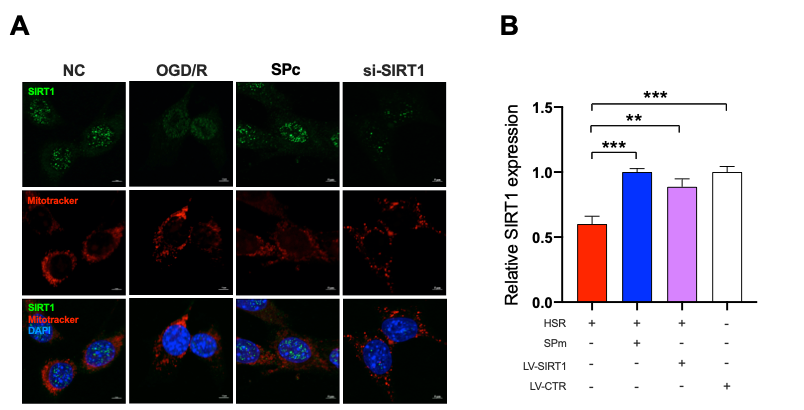


Figure S1. Sevoflurane postconditioning varied the deposition and distribution of SIRT1 following IRI.

By using immunofluorescent staining, the distribution of SIRT1 were presented in the HT22 cells (scale bar = 5 µm), and quantified in the hippocampal tissues following IRI. Sevoflurane postconditioning was performed at the beginning of reoxygenation in HT22 cells (SPc) or at the onset of blood refusion in mice (SPm). During OGD/R, transfection of siRNA against Sirt1(si-SIRT1) was introduced 24 hours before OGD/R with SPc. Nonsense sequence for SIRT1 was the negative control (NC). Interestingly, SIRT1 accumulated in the nucleus before IRI or with SP. IRI significantly reduced the SIRT1 expression. After overexpressing SIRT1 through lentivirus transfection, mice were exposed to HSR without SPm. Control lentiviral vector (LV-CTR) was cerebrally injected without HSR or SPm. LV-SIRT1 effectively reversed the SIRT1 loss induced by HSR. Mitochondria was labelled with Mitotracker. Data were analyzed by one-way ANOVA followed by the Tukey *post hoc* test. All values are represented as mean ± SEM., n = 3 per group, ***p* < 0.01, ****p* < 0.001.


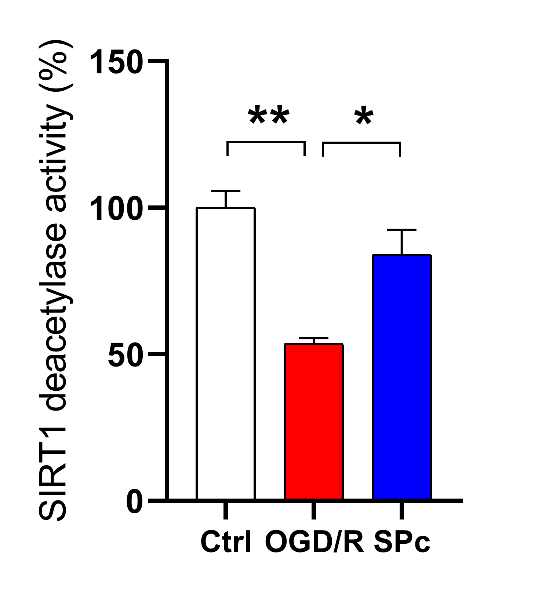


Figure S2. Relative deacetylation activity of SIRT1 after sevoflurane postconditioning in HT22 cells. To detect the deacetylation activity of SIRT1 in the nucleus, nuclear proteins were conducted to the SIRT1/Sir2 deacetylase fluorometric assay kit Ver.2. Data were analyzed by one-way ANOVA followed by the Tukey *post hoc* test. All values are represented as mean ± SEM., n = 3 per group, **p* < 0.05, ***p* < 0.01**.**

Figure S3. Relative mRNA level of SIRT1 after siRNA transfection in HT22 cells. Nonsense sequence for SIRT1 was the negative control (NC). The silence efficacy was determined by quantitative real-time polymerase chain reaction. Data were analyzed by one-way ANOVA followed by the Tukey *post hoc* test. All values are represented as mean ± SEM., n = 3 per group, *****p* < 0.0001**.**

Figure S4. Sevoflurane postconditioning promoted the cell viability following OGD/R.

Oxygen and Glucose Deprivation/Reoxygenation (OGD/R) was a model of IRI *in vitro*. Sevoflurane postconditioning (SPc) was performed at the beginning of reoxygenation in HT22 cells. Data were analyzed by one-way ANOVA followed by the Tukey *post hoc* test. All values are represented as mean ± SEM., n = 3 per group, ****p* < 0.001, *****p* < 0.0001**.**

Figure S5. Sevoflurane postconditioning attenuated the apoptosis induced by OGD/R.

By using double-labelling immunofluorescent staining, relative quantifications of the fluorescent intensities of cleaved caspase 3 (left) and TUNEL (right) were presented. Sevoflurane postconditioning (SPc) was performed at the beginning of reoxygenation in HT22 cells. During OGD/R, transfection of siRNA against Sirt1 (si-SIRT1) was introduced 24 hours before OGD/R with SPc. Nonsense sequence for SIRT1 was the negative control (NC). All quantitative analyses were performed from at least three independent experiments. Immunofluorescent intensity was analyzed with Image J software. Data were analyzed by one-way ANOVA followed by the Tukey *post hoc* test. All values are represented as mean ± SEM., n = 3 per group, ****p* < 0.001, *****p* < 0.0001**.**


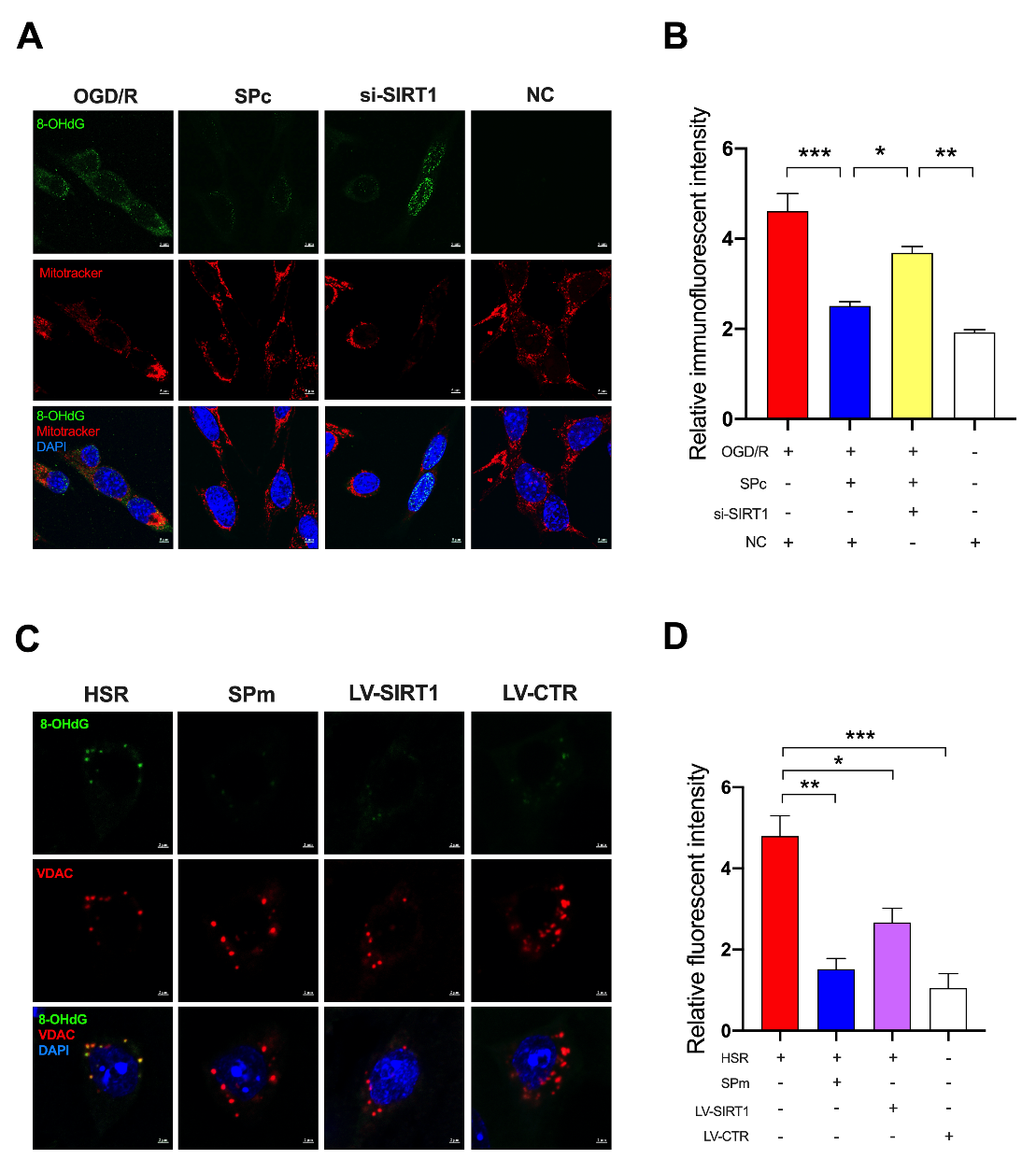


Figure S6. Sevoflurane postconditioning attenuated DNA damage induced by OGD/R.

By using immunofluorescent staining, the distributions of 8-OHdG were presented and quantified in the HT22 cells (scale bar = 5 µm) and hippocampal tissues (scale bar = 2 µm) following IRI (B, *F* = 19.39, *p* = 0.0005; D, *F* = 31.42, *p* < 0.0001). Notable DNA damage was consistently observed in two IRI models. Sevoflurane postconditioning significantly attenuated the nuclear accumulation of 8-OHdG in both HT22 cells and hippocampi. Sevoflurane postconditioning was performed at the beginning of reoxygenation in HT22 cells (SPc) or at the onset of blood refusion in mice (SPm). During OGD/R, transfection of siRNA against Sirt1 (si-SIRT1) was introduced 24 hours before OGD/R with SPc. Nonsense sequence for SIRT1 was the negative control (NC). After overexpressing SIRT1 through lentivirus transfection, mice were exposed to HSR without SPm. Control lentiviral vector (LV-CTR) was cerebrally injected without HSR or SPm. Mitochondria was labelled with Mitotracker or VDAC. Data were analyzed by one-way ANOVA followed by the Tukey *post hoc* test. All values are represented as mean ± SEM., n = 3 per group, ****p* < 0.001, *****p* < 0.0001**.**
